# The temporal context of eye contact influences perceptions of communicative intent

**DOI:** 10.1098/rsos.250277

**Published:** 2025-07-16

**Authors:** Nathan Caruana, Friederike Charlotte Hechler, Emily S. Cross, Emmanuele Tidoni

**Affiliations:** ^1^ Flinders University Institute for Mental Health and Wellbeing, Adelaide, South Australia, Australia; ^2^ College of Education Psychology and Social Work, Flinders University, Adelaide, South Australia, Australia; ^3^ Department of Psychological Sciences, Macquarie University, Sydney, New South Wales, Australia; ^4^ Universität Potsdam, Potsdam, Brandenburg, Germany; ^5^ ETH Zurich, Zürich, Switzerland; ^6^ Department of Psychology, University of Leeds, Leeds, UK

**Keywords:** eye contact, joint attention, communication

## Abstract

This study examined the perceptual dynamics that influence the evaluation of eye contact as a communicative display. Participants (*n* = 137) completed a task where they decided if agents were inspecting or requesting one of three objects. Each agent shifted its gaze three times per trial, with the presence, frequency and sequence of eye contact displays manipulated across six conditions. We found significant differences between all gaze conditions. Participants were most likely, and fastest, to perceive a request when eye contact occurred between two averted gaze shifts towards the same object. Findings suggest that the relative temporal context of eye contact and averted gaze, rather than eye contact frequency or recency, shapes its communicative potency. Commensurate effects were observed when participants completed the task with agents that appeared as humans or a humanoid robot, indicating that gaze evaluations are broadly tuned across a range of social stimuli. Our findings advance the field of gaze perception research beyond paradigms that examine singular, salient and static gaze cues and inform how signals of communicative intent can be optimally engineered in the gaze behaviours of artificial agents (e.g. robots) to promote natural and intuitive social interactions.

## 1. Introduction

Social gaze is an important source of information in guiding how we understand and interact with others [1]. During the pre-language era of human evolution, the ability to signal and perceive gaze cues from conspecifics is believed to have been critical in supporting collaboration and communication [2]. For example, perceiving others’ gaze can inform about another person’s locus of attention to guide joint attention—our ability to attend to the same things as others [3]. Gaze-led joint attention is pivotal during human infancy for supporting both language and social cognition development [4,5]. Furthermore, adults implicitly and effectively attend to and integrate gaze signals to facilitate joint attention during dyadic interactions, even when more explicit gestures (e.g. hand pointing) are being used to communicate joint attention bids [6,7].

Eye contact (i.e. direct gaze) is a particularly important communicative signal, with the capacity to rapidly capture attention [8,9]. Observing direct gaze also modulates the activation of neural substrates associated with making inferences about the perspectives and intentions of others [10]. The influence of eye contact on social-cognitive processing has been broadly referred to as the ‘Eye contact effect’ and, according to the ‘Fast track modulator’ model, observing direct gaze engages neural mechanisms that have evolved to rapidly process and execute responses to face-bound social cues [11]. This involves subcortical pathways (e.g. superior colliculus, pulvinar and amygdala) that likely trigger downstream social-cognitive processes (e.g. perspective taking [11]).

Eye contact is considered a special social signal for conveying a conspecific’s readiness or intention to communicate (see [10] for review). Eye contact displays have been found to increase the likelihood of conversation initiation [12], and promote gaze-following when averted gaze shifts follow eye contact observed from a second-person [13,14] and third-person perspective (i.e. observing mutual gaze between two other agents [15]). This has been presented as evidence that the act of engaging in or observing eye contact increases the social relevance of subsequent eye movements. Such accounts are also indirectly supported by neuroimaging studies, which show that observing eye contact—particularly during coordinated interactions—modulates activation in neural substrates associated with the ‘Theory-of-Mind network’, including the medial prefrontal cortex, superior temporal sulcus and temporal parietal junction [16–18]. In a series of behavioural studies, Caruana and colleagues have argued that eye contact cues are critical during gaze-based joint attention interactions, as they help differentiate communicative gaze shifts from non-communicative gaze shifts displayed during a visual search [19,20]. More recently, Alhasan & Caruana [21] provided evidence suggesting that eye contact helps to rapidly differentiate gaze shifts that are communicative (i.e. those that immediately follow eye contact to signal a joint attention bid) from those that are unlikely to be communicative (i.e. those displayed by an agent before making eye contact to privately search for a joint attention target). However, no study, to our knowledge, has yet investigated whether the perception of communicative intent can be *modulated* by the temporal sequence and frequency of eye contact in dynamic gaze sequences. This line of enquiry is critical for elucidating precisely *how* the perceptual dynamics of eye contact can modulate perceived communicativeness during social interactions.

### 1.1. Current study

**Figure 1.**
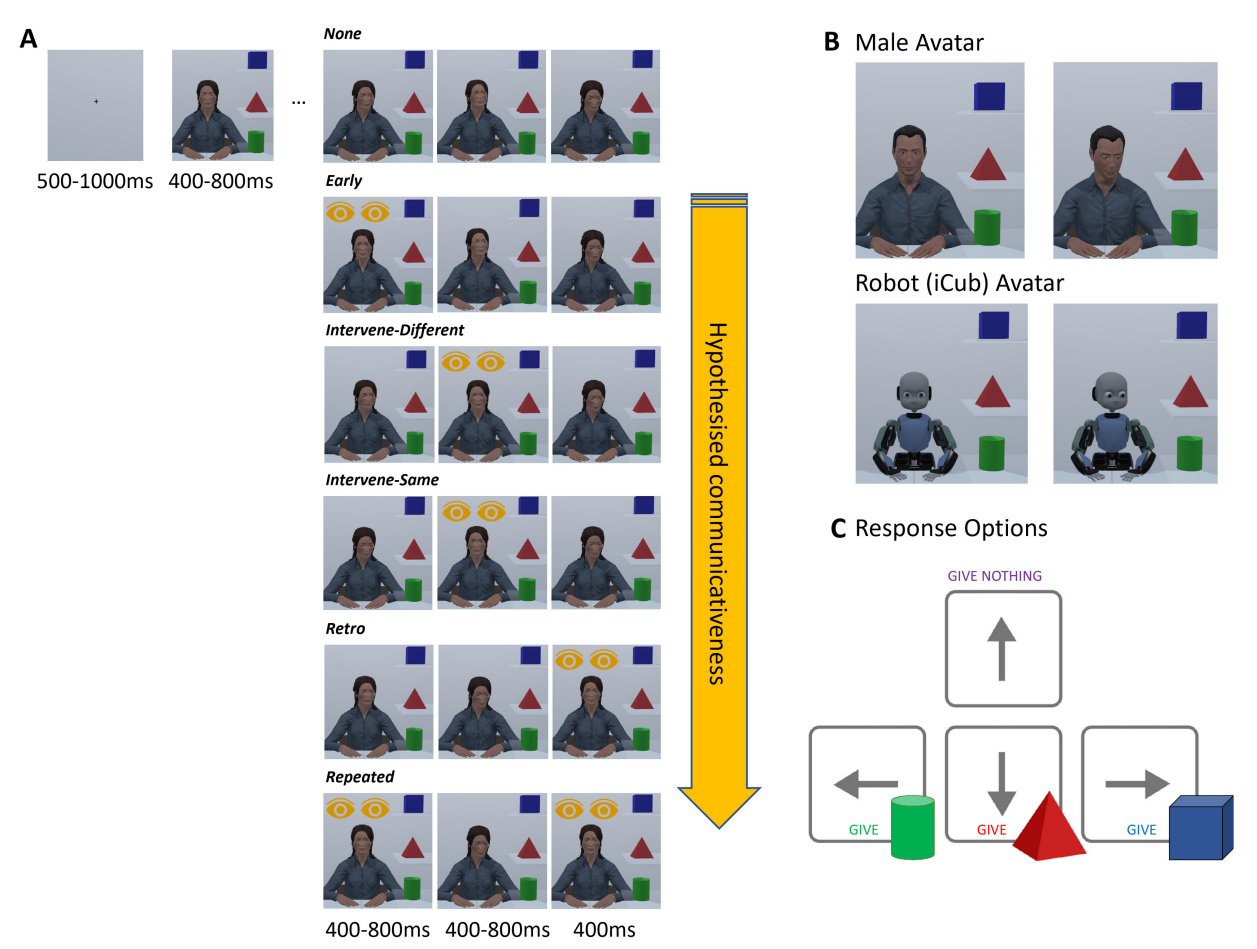
(A) Trial sequence examples for all six gaze conditions. The yellow eye icons were not part of the experimental stimuli but are depicted here to highlight when direct gaze was displayed in each condition. We hypothesized that sequences with more instances of direct gaze, and/or direct gaze displayed in closer temporal proximity to the final gaze shift would be perceived as more communicative than instances with fewer or no opportunities for perceived eye contact; indicated by the yellow arrow icon. In one block, participants completed the task with an anthropomorphic agent avatar as their partner, counterbalancing female (A) and male (B) avatars across the full sample. In another block, participants interacted with a robot agent resembling an animated version of the iCub robot (https://icub.iit.it/) (B). Blocks appeared in counterbalanced order across participants. In each trial, participants decided whether to ‘give’ the agent one of the three blocks or nothing at all, using the arrow keys on a standard keyboard (C).

To simulate gaze evaluation in face-to-face interactions, we developed a second-person perspective collaborative task in which participants completed a game with an on-screen (human or robotic) agent (cf. [22,23]). Participants observed an agent sitting behind a table with a shelf on the right side of the screen containing three different objects (see figure 1). Participants were told that, in each trial, the agent needed one of the three blocks to complete the construction of an unseen block model. In some trials, the required block would be available to the agent, but, in others, the agent required the participant to share the block.

In each trial, the agent gazed three times at the objects and participants had to decide at the end of the third gaze shift whether to ‘give’ one of the blocks to the agent (i.e. indexing the perception of a communicative request for one of the blocks), or ‘give nothing’ (i.e. indexing the perception that the agent is not communicating a request for assistance, and is privately searching the blocks).

We manipulated the temporal sequence and frequency of eye contact across six conditions (see figure 1) to determine the perceptual features of eye contact that influence whether an agent’s behaviour is evaluated as signalling a communicative intention. Overall, we anticipated that the temporal proximity of eye contact (i.e. the recency of the display in a sequence of eye movements), and the frequency of eye contact displays within that sequence, would be important variables for increasing the potency of communicative signals. That is, we expected participants would be more likely to ‘give’ blocks (i.e. perceive communicative intent) in conditions where eye contact occurred closer to the final gaze shift, and when there were multiple instances of eye contact before and after an averted gaze shift at the final block location [24]. We visualize the specific direction of this hypothesis across our six conditions in figure 1A, as well as in table 1, below. Our method and hypotheses were pre-registered on the Open Science Framework (https://osf.io/w68ut/).

**Table 1.** Eye contact sequence manipulations across conditions and predicted communicativeness.

gaze condition	temporal sequence of eye contact	multiple eye contact displays	predicted communicativeness
**none**	N/A	no	least
**early**	eye contact precedes	no	moderate
**intervene-different**	eye contact between gaze shifts to different locations	no	moderate
**intervene-same**	eye contact between gaze shifts to the same location	no	high
**retro**	eye contact follows	no	high
**repeated**	eye contact precedes and follows	yes	highest

*Note*. Gaze conditions are listed in order of hypothesized communicativeness from top (least communicative) to bottom (most communicative).

A secondary aim of this study was to examine whether the influence of eye contact on the perception of communicative intent generalizes across interactions with human and humanoid robot agents. Previous studies examining gaze processing (e.g. using Posner-style cueing paradigms) have observed robust gaze cueing effects in non-anthropomorphic stimuli—including schematic and robotic face [25–27]. However, recent work from our group found distinct within-subjects effects based on the appearance of an agent—irrespective of whether they believed the agent to be human- or AI-controlled [28]. Specifically, using the same agent stimuli depicted in figure 1, we found that participants were significantly more likely to look at the humanoid robot than the human face during a hand-cued joint attention task where the agent’s eyes naturally moved throughout the interaction. However, participants were faster to respond to hand-cued joint attention bids displayed by the human avatar than the robot agent [28]. Across several other experiments, we have also found differences in the evaluation of gaze and intentions from human and robotic agents, with evidence that human gaze, but not robot gaze, facilitates action prediction, with faster overall inferences made about the mental states of agents that appear as humans rather than robots [29,30]. Taken together, existing evidence suggests that while our sensitivity to gaze cues may be broadly tuned, enabling gaze-related effects to manifest when displayed by agents of varying degrees of humanness, this may be attenuated depending on the observed agent, interactive context or behavioural outcome. Confirming whether the perceptual features of eye contact displays influence perceptions of communicative intent, across both human and non-human agents, is important for determining the extent to which findings can be used to inform the design of communicative behaviour among social robots. Findings from the current study will also inform the extent to which theories of gaze processing, including the fast track modulator model, explain gaze perception across diverse gaze stimuli, including artificial and robotic agents.

## 2. Methods

### 2.1. Participants

We pre-registered our intention to recruit and test 156 participants via the Prolific Academic online research participation platform [31]. Prior to data collection, we conducted an *a priori* power analysis using G*Power v3.1 [32] to determine the minimum sample size required to detect a within-subjects effect of interest (i.e. for the main effect of Gaze). We assumed a Cohen’s *d* = 0.3 as a conservative estimate of the smallest effect size of interest, with a significance level (*α*) of 0.05 and power of 90%. This analysis indicated a minimum required sample size of *n* = 119 for detecting differences in pairwise comparisons between gaze conditions. Given the complexity of our design and the exploratory nature of some comparisons (e.g. Agent × Gaze interactions), power analyses focused on the primary effect of interest only (i.e. Gaze). Furthermore, we conservatively oversampled to *n* = 156 to account for potential data loss due to exclusions or missing responses.

To further validate the adequacy of our sample size, we additionally conducted a series of retrospective power analyses using the Superpower R package [33], which allows for simulation-based power estimation in factorial designs. The simulations were based on the minimum sample size derived from our pre-registered power analysis (*n* = 119), assuming a within-subjects 2 (Agent: Human, Robot) × 6 (Gaze) design. These simulations modelled three different plausible scenarios involving different patterns of effects across gaze and agent conditions. For example, assuming all gaze conditions differ from each other with an equivalent effect size of *d* = 0.3, and that robot agents produce more attenuated gaze effects than human agents (also equivalent to *d* = 0.3). Under such a simulation, a sample of *n* = 119 yielded >99% power to detect main effects of Gaze and Agent. Another simulation estimated >95% power to detect a significant Gaze*Interaction effect. These simulations are summarized in detail in Supplementary Power Analysis document on the project’s OSF project page, along with the R code used for each simulation (https://osf.io/w68ut/).

Participants were reimbursed £9.50 per hour for their participation in this study, which lasted 37 mins in total, on average. Participants were eligible to enrol in the study if they were at least 18 years of age and could read proficiently in English. The sampled participants were ethnically diverse but underrepresented Asian people (White: 53.21%; Black: 33.33%; Mixed: 7.62%: Other: 5.13%; Asian: 0.64%). Most participants were engaged in either full-time (43.66%) or part-time work (19.72%) and 47.45% of participants indicated that they were currently students.

Data collection was completed in four batches to allow for a balanced sampling of males and females who were assigned to complete the task with either a male or female human-like avatar. Participants who identified as non-binary or preferred not to say could take part in any batch. Participants who identified as trans-male could take part in the two male batches. Participants who identified as trans-female could take part in the two female batches.

Following data pre-processing and data exclusion (see §2.4 below), we had a final sample size of *n* = 137 (see table 2 for a summary of *Participant Characteristics*).

**Table 2.** Participant characteristics. *Note*. Age and CATI scores reported by gender, with mean and standard deviation reported in the format *M*(SD). *Age and gender were not disclosed for this one participant.

	*N*	%	age	CATI (total)	CATI (social interaction)	CATI (communication)
**gender**						
***female***	63	45.9	27.60 (7.59)	129.62 (21.58)	22.81 (7.21)	15.56 (4.45)
***male***	67	48.9	27.91 (8.44)	125.30 (22.59)	20.08 (7.12)	15.78 (4.25)
***non-binary***	6	4.38	26.67 (4.68)	133.67(11.11)	25.00 (4.29)	15.17 (1.84)
***undisclosed***	1	0.73	*	123.00 (NA)	32.00 (NA)	9.00 (NA)
**total**	137	NA	27.71 (7.89)	127.64 (21.70)	21.64 (7.22)	15.60 (4.27)

To characterize the sample—and to support our pre-registered exploratory analyses (see https://osf.io/w68ut/)—participants completed an online version of the Comprehensive Autistic Trait Inventory (CATI [34]), which measures variation in subclinical autistic traits (see table 2). The CATI was completed after the experimental task and comprised one attention check. Five participants failed the attention check, and their data were excluded from exploratory analyses that involved CATI data. In line with our pre-registration, we explored whether the CATI total score and subscale scores for *Communication* and *Social Interaction* correlated with behavioural data (see below), including ‘Give’ frequencies and reaction times. We found no notable correlations (all *p*s > .046)^1^. All exploratory analyses involving the CATI—including descriptive statistics—are summarized in a separate RMarkdown on our study OSF project page (https://osf.io/w68ut/).

### 2.2. Ethics statement

All participants provided informed consent before completing the study, which was carried out in accordance with the protocol reviewed and approved by the University of Hull’s Human Ethics Committee (protocol number: FHS467).

### 2.3. Task stimuli and experimental design

#### 2.3.1. Experimental task

In a novel semi-interactive task participants ‘assisted’ a virtual partner to complete the construction of an unseen block model in each trial. Participants were told that the partner must select one of three blocks visible on the screen (figure 1). Sometimes these blocks would be available to the agent, and sometimes the agent required the participant to ‘give’ them the required block. In each trial, participants made a judgement as to whether their partner was intentionally signalling a communitive request and, thus, whether they should ‘give’ the agent one of the three blocks, or ‘give nothing’, via four keyboard responses (figure 1C). The collaborative context enabled perceptions of communicative intent to be indexed via ecologically valid social responses, rather than participants having to make explicit social judgments about the agent’s eye gaze behaviour from a third-person (i.e. non-interactive and observational) perspective [23]. However, participants were not deceived into believing that they were interacting with a genuine human partner in real time. Moreover, details about the agents’ sentience or intelligence were not specified in any way. The precise visual instructions presented to participants in this study are provided, alongside experiment code in PsychoPy https://www.psychopy.org/) [35] and all associated data and analyses, on our Open Science Framework (OSF) project page (https://osf.io/w68ut/).

#### 2.3.2. Task stimuli

In each trial, participants saw a virtual human (male or female counterbalanced across participants, see §2.1) or robot agent, modelled after the iCub robot; https://icub.iit.it/; see figure 1B). Human avatar stimuli were developed in a previous study and were specifically designed and evaluated to be ethnically ambiguous [6]. Avatars were adapted in Unity Game Engine to manipulate the agent’s gaze when averted towards the three blocks, which were also embedded in the virtual environment using Unity Game Engine. All task stimuli and experimental code can be found on our OSF project page (https://osf.io/w68ut/).

The human agent stimuli (figure 1A,B) have previously been validated in several virtual reality studies in which participants have evaluated them to be human-like and effective in simulating natural, cooperative and intuitive social interactions [6,7]. Further, these stimuli have been shown to validly interrogate how the perceptual properties of dynamic gaze influence joint attention during genuine and simulated virtual interactions ([28]; see also [22] for a discussion on the valid use of animated virtual agents for studying gaze dynamics). The robot agent stimuli have also been previously validated in an immersive virtual reality study, where we found evidence for significant differences in the way people perceived the human and robotic agents [28]. People rated the robotic agent as significantly less human-like than the human agent with respect to its appearance and eye-movement behaviour. This demonstrates that these stimuli are appropriate for examining possible differences in how the perceptual dynamics of gaze perception may (or may not) differ between human and robotic social agents. The agent appeared across trials, seated behind a table with both hands resting on the tabletop. To the right of the agent, there were three transparent shelves displaying a green cylinder (bottom shelf), a red pyramid (middle shelf) and a blue cube (top shelf). The location of the three blocks remained static throughout the duration of the experiment. Each trial began with a central fixation cross displayed for 500−1000 ms, followed by the presentation of the agent, which was initially displayed with their eyes closed. The centre of the agent’s face was aligned with the location of the preceding fixation cross. The eye gaze of the agent then updated three times, by displaying a sequence of static images to create the perception of apparent motion in the agent’s gaze behaviour. The delay between each stimulus presentation (i.e. agent appearing after the fixation cross and the first two gaze changes) was randomized across a distribution of 400−800 ms. This jittered presentation was used to add realistic variability in the agents’ gaze behaviour. The final gaze shift was displayed for a standard 400 ms before participants were prompted to give their response. This was to ensure that the viewing time for the final gaze shift was standardized across participants to minimize any impact this might have on reaction time measures. This approach towards simulating realistic dynamic gaze behaviour is consistent with previous interactive studies of gaze-based joint attention [16,19,20]. During the response phase at the end of each trial, participants were shown a visual summary of the response-key mappings to minimize memory demands during the task (figure 1C). Participants were instructed to answer as quickly as possible.

#### 2.3.3. Experimental design

We manipulated the frequency and temporal sequence of eye contact displays across six conditions (see figure 1): (1) *No Eye Contact*: the agent displayed three averted gaze shifts to each of the three blocks; (2) *Early Eye Contact*: the agent immediately made eye contact upon opening their eyes, before averting their gaze twice and look at two of the blocks; (3) *Eye Contact Intervening Gaze Shifts in Different Directions*: the agent averted their gaze to one block, made eye contact and then averted gaze towards a second block; (4) *Eye Contact Intervening Gaze Shifts in Same Direction*: the agent averted their gaze to one block, made eye contact and then averted gaze towards the same block; (5) *Retrospective Eye Contact*: the agent averted gaze to two different blocks and then made eye contact; and (6) *Repeated Eye Contact*: the agent made eye contact, averted their gaze towards a block and then made eye contact again. For simplicity, we have abbreviated the conditions to the following respective labels: (1) None; (2) Early; (3) Intervene-Different; (4) Intervene-Same; (5) Retro; and (6) Repeated. For each Gaze Sequence condition, there were six unique trials in which we carefully counterbalanced the order and combinations of averted gaze shifts towards each of the block locations.

In addition to Gaze Sequence, we manipulated whether people observed a human-like avatar or a robotic avatar across two task blocks, with agent order counterbalanced across participants. This resulted in a 2 (Agent: Robot, Human) × 6 (Gaze: see above) fully-within-subjects design. Agent conditions were completed as blocks in counterbalanced order. As noted above, we counterbalanced, across participant gender groups, whether they interacted with a male or female human agent. Those who identified as non-binary, or who did not disclose their gender, were randomly allocated to interact with the male or female agent.

The Human and Robot task blocks comprised a total of 288 trials; six gaze conditions, each comprising six unique trials that were repeated four times across the block (6 conditions × 6 unique trials × 4 repetitions = 144 trials per agent). Each block was divided into three sessions allowing participants to take two self-paced breaks. Trial order was randomized. The entire experimental task took approximately 25 min to complete.

#### 2.3.4. Procedure

Upon providing informed consent, participants navigated through a series of written instructions at their own pace (see the project OSF page for a complete set of task instructions; https://osf.io/w68ut/). Then, before commencing the experimental task, participants were provided with the opportunity to practice the response-key mapping across eight trials (two trials per response key). This practice did not take the form of a complete experimental trial. Participants were simply asked to press the key that corresponded with a specified response, such as ‘give nothing’, ‘give cylinder’, etc. Participants received feedback in each of these trials. This ensured participants were familiar with the response options before commencing the task.

Participants then completed the two main blocks with the Human and Robot agent in counterbalanced order. At the end of each block, participants completed the Godspeed scales [36] with reference to the respective agent they just observed. The scales are primarily used in human–robot interaction research and comprise items that are designed to capture a person’s perception of an agent across five key domains: *Anthropomorphism*, *Animacy*, *Likeability*, *Perceived Intelligence* and *Perceived Safety*. As outlined in our pre-registration, this enabled us to characterize and explore whether any observed interaction between agent type and eye contact effects on perceived communicativeness aligned with individual differences in agent perception and subjective experience. Finally, at the very end of the experimental task, participants completed the CATI (as described in §2.1, above).

### 2.4. Data and statistical analysis

All analysis code is documented and shared alongside all raw data and our pre-registration on this study’s OSF project page (https://osf.io/w68ut/).

#### 2.4.1. Pre-processing data

We first inspected data for evidence of acquiescent response styles during the task. We did this by examining the distribution of participants’ ‘Give’ responses across the three block objects (left/downward/right arrow keys). Since the task was designed to counterbalance the block location that the agent gazed towards last, we expected ‘Give’ responses to be equally distributed across the three ‘Give’ response keys, with an SD of response frequency close to zero. Seven participants had SDs that were two SDs greater than the average SD observed across these three response options (i.e. >29.62). This deviation indicates a divergent preference to ‘give’ a particular block significantly more (or less) than the others, and possibly an acquiescent response strategy to expedite study participation. This follows in that if a participant is engaging in the task, we should observe an approximately equal proportion of ‘Give’ responses for each block key (i.e. for cube, pyramid and cylinder). An even split between these three types of responses should result in an SD close to 0 (i.e. no variation in response rate across the three blocks). Larger SDs would indicate that the participant favoured one block over another, and thus, a possible acquiescent response pattern. Following the above criteria, seven participants were excluded from all analyses. We did not originally pre-register this initial data exclusion step, but implemented it retrospectively when we observed that some participants showed some evidence of acquiescent responding. We believe this additional step offers a conservative approach to data analysis. We have also confirmed that the pattern of results remained the same whether these participants were excluded or retained in analyses.

Next, we removed trials with excessively short or long reaction times (RTs). This was defined in our pre-registration as RTs < 150 ms and >3000 ms from the onset of the response prompt at the end of the trial, as these were likely to be pre-emptive or ‘guess’ responses. This resulted in the removal of 4.66% of trials from the dataset. Based on the remaining trials, we computed how many illogical responses participants provided across trials, indicative of a random response style. There were no objectively ‘correct’ or ‘incorrect’ responses in our experimental task. However, in line with our pre-registration, we defined an illogical response as a ‘Give’ response for an object that the agent never gazed towards during a particular trial. Note that this procedure was not possible in the *None* (i.e. no eye contact) condition since the agent looked at all three objects in these trials. We removed eight participants who had illogical response rates that were two SDs greater than the sample mean (i.e. illogical response rate >49.06%). We then removed all trials with illogical responses from the remaining dataset, resulting in the removal of 10.10% of remaining trials. Finally, we removed five participants whose average RTs were more than two SDs greater than the sample mean (i.e. mean RT < 325.78ms or >1089.21 ms). This resulted in a final sample size of *n* = 137. Table 3 summarizes the average number of trials retained for each condition and response type (i.e. Give versus No Give) after data exclusion. All remaining participants were included in analyses using linear mixed-effects modelling (see below for more details). We originally pre-registered to conduct ANOVAs on aggregated data. However, for seven participants in the remaining sample, data was missing from at least one condition (e.g. due to excessive errors, or because they never provided a ‘Give’ or ‘No Give’ response for one or more condition). As such, for analyses using aggregated participant data, we had a total sample size of *n* = 130 (see below for further details).

**Table 3.** Mean trial count after data preprocessing by condition and response type.

		‘give’		‘no give’		total	
		*M*	SD	*M*	SD	*M*	SD
**human**	***none***	5.59	8.00	17.70	8.23	23.29	1.99
	***early***	7.75	8.72	11.36	9.83	19.12	7.68
	***intervene-different***	11.85	9.37	6.99	8.73	18.84	7.94
	***intervene-same***	20.72	4.18	0.85	2.34	21.57	3.52
	***retro***	10.11	8.37	8.00	8.20	18.11	7.03
	***repeated***	20.20	5.43	2.19	4.94	22.39	2.68
**robot**	***none***	6.18	8.28	17.04	8.32	23.21	1.92
	***early***	7.77	8.60	11.20	9.55	18.96	7.83
	***intervene-different***	12.43	9.46	6.56	8.65	18.99	7.80
	***intervene-same***	21.53	3.27	0.82	1.97	22.35	2.38
	***retro***	10.41	8.87	7.83	8.42	18.24	7.43
	***repeated***	20.42	5.63	2.37	5.24	22.79	2.16

#### 2.4.2. Statistical analyses

Our pre-registration outlined a plan to conduct ANOVAs on aggregated data for our primary analysis, focused on comparing ‘Give’ frequencies, calculated as percentages per conditions, as well as a secondary exploratory analysis comparing RTs across conditions. Post hoc comparisons were carried out for significant main effects and interactions, implementing a Holm Family-Wise Error correction for multiple comparisons.

Upon inspecting the collected data, we discovered that ANOVAs using aggregated data were not entirely appropriate for the RT analysis, given that some participants did not always contribute a ‘Give’ or ‘No Give’ response within each condition (as described above). As such, ‘null’ RT data for some participants and conditions would present a bias in the aggregated RTs if they were coded as 0 (reducing the average RT estimates) or as missing (resulting in participant observations being removed entirely from some conditions but not others). We therefore implemented linear mixed-effects (LME) models for the exploratory RT analysis (i.e. those that were not pre-registered) as well as the ‘Give’ frequency analysis, alongside the pre-registered ANOVA analyses on aggregated data. RT analyses included the full sample (*n* = 137), whereas the pre-registered ANOVA analyses on aggregated data only included participants who contributed data to each condition and response type (*n* = 130).

LME models address the problem of missing data because they treat each trial, rather than each subject as a unique observation. Additionally, they can more robustly account for random effects, including those attributable to variation across trials within conditions and across participants. LME models were estimated using the maximum likelihood estimation method within the *lme4* R package [37]. Adding LME models also offered the additional benefit of corroborating ANOVA analyses while accounting for subject and trial-level variance (i.e. random effects) when estimating the fixed effect parameters of interest (i.e. Gaze Condition, Agent Type). In line with recommendations for implementing mixed random-effects models, we attempted to implement a ‘maximal’ random factor structure for both analyses. However, given the complexity of the required random effects structure, estimating these saturated models was not always possible using the available data, resulting in either a singular fit or convergence failures [38]. We therefore implemented the LME analysis pipeline, proposed by Scandola & Tidoni [39], by fitting complex random intercepts (CRI) models via the afex package ([40]; CRAN: Package afex), a wrapper of lme4 ([41]; CRAN: Package lme4), using the Satterthwaite degrees-of-freedom approximation [42]. Specific model selection details are summarized below when reporting results and are also reported extensively in the accompanying RMarkdown documentation on our project OSF page (https://osf.io/w68ut/). A significance criterion of *α* < 0.05 was employed and Post hoc comparisons were conducted using the *emmeans* package [43] and a Holm correction implemented to account for multiple comparisons.

Finally, to account for the positive skew typically observed in reaction time (RT) data, we applied a Box-Cox transformation prior to statistical analysis, using the boxcox() function from the **MASS** package in R. This transformation identifies an optimal lambda (*λ*) to reduce skewness and better satisfy the assumptions of linear modelling.

## 3. Results

### 3.1. ‘Give’ versus ‘No Give’ responses

Our primary pre-registered analyses focused on evaluating whether there were any differences in the tendency to ‘give’ (i.e. perceive communicativeness) across the gaze conditions, and whether any such differences were modulated by the human- or robot-like appearance of the agent. Descriptive statistics for the percentage of trials in which participants made a ‘Give’ response are summarized in table 4.

**Table 4.** ‘Give’ frequency descriptive statistics by condition and agent.

gaze condition	agent	mean (%)	SD
**none**	***human***	25.76	35.13
	***robot***	27.82	35.90
**early**	***human***	41.32	40.20
	***robot***	41.89	39.30
**intervene-different**	***human***	62.50	39.24
	***robot***	64.47	38.54
**intervene-same**	***human***	95.76	10.99
	***robot***	96.01	9.38
**retro**	***human***	55.97	38.30
	***robot***	57.37	38.66
**repeated**	***human***	89.84	21.50
	***robot***	88.99	22.85

*Note*. ‘Give’ frequencies are summarized as the percentage of trials that participants responded by giving a block to the agent.

#### 3.1.1. ANOVA on aggregated data

**Figure 2.**
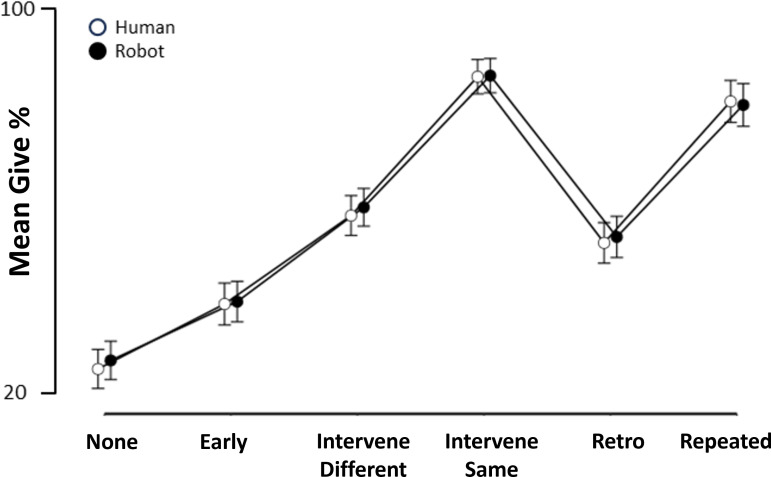
Frequency of ‘Give’ responses by Gaze condition, for both Agent (Human, Robot) conditions. Error bars represent 95% confidence intervals.

**Figure 3.**
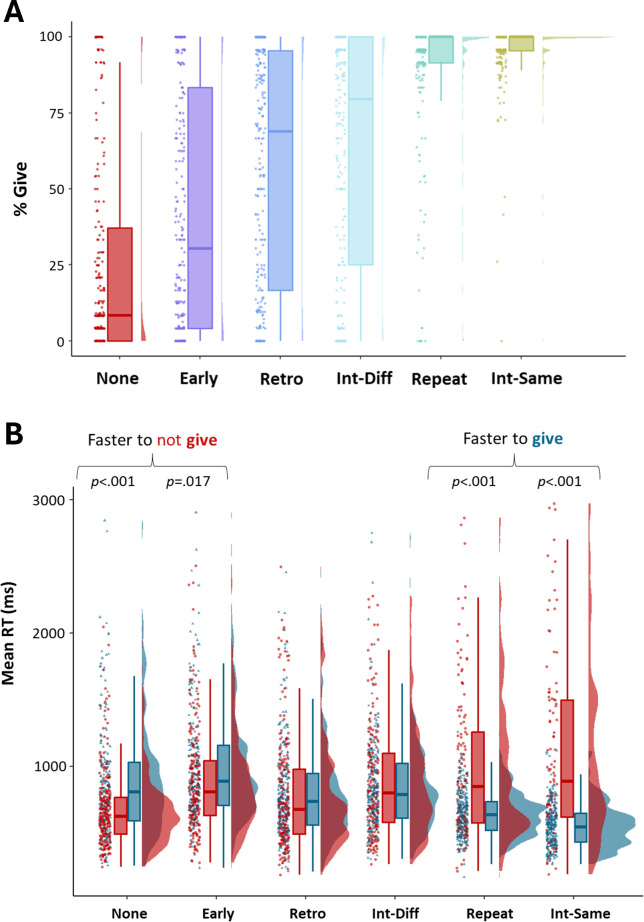
(*A*) Frequency of ‘Give’ responses by Gaze conditions. (*B*) Reaction time to ‘Give’ and ‘No Give’responses by Gaze condition. Data in both plots are averaged across Agent (Human, Robot) conditions. Data are summarized with individual data points, with conditions arranged in observed order of increasing communicativeness from left to right.

An ANOVA on ‘Give’ frequency data revealed a significant main effect of Gaze condition (*F*_(2.96, 382.35)_ = 135.65; *p* < .001; η²_p_ = .513, Greenhouse-Geisser corrected) but with no evidence of a main effect of Agent (*F*_(1,129)_=.819, *p* = .367, *η²_p_ =* .006), nor an Agent * Gaze interaction (*F*_(3.73, 481.51)_ = 0.47, *p* = .746, _η²p_ = .004, Greenhouse-Geisser corrected). We conducted a Bayesian ANOVA to further interrogate the absence of significant Agent effects, which revealed strong to extreme evidence for no effect of Agent Type and associated interactions. Specifically, we found strong evidence for the null hypothesis when comparing a model comprising the Agent factor alone to the null model (BF_10_ = .062, Error = 1.71%). Further, compared with a model comprising the Gaze factor (i.e. the best model), we found strong evidence for the null hypothesis when adding the Agent factor to the model (i.e. Gaze + Agent; BF_10_ = .068, Error = 1.82%), and extreme evidence for the null hypothesis when adding the interaction term (i.e. Gaze +Agent + Gaze * Agent; BF_10_ < .0001, Error = 1.28%). Together, this evidenced commensurate Gaze effects across the Human and Robot agent conditions. As summarized in table 5, Holm-corrected post hoc comparisons revealed significant differences between all Gaze condition pair-wise comparisons (*p* < .003), revealing that *Intervene-Same* was perceived as the most communicative, followed by *Repeated*, *Intervene-Different*, *Retro*, *Early* and then *None,* which had the lowest rate of ‘Give’ responses than any other condition. This pattern was consistent across both agent conditions (figure 2). Frequency data collapsed across agent is visualized in figure 3A.

**Table 5.** Post hoc comparisons for Gaze condition effect.

		*t*	Cohen’s *d*	*p*_holm_
**none**	***early***	−7.459	−0.654	<.001
	***intervene-different***	−11.506	−1.009	<.001
	***intervene-same***	−20.980	−1.840	<.001
	***retro***	−8.552	−0.750	<.001
	***repeated***	−15.711	−1.378	<.001
**early**	***intervene-different***	−7.085	−0.621	<.001
	***intervene-same***	−15.418	−1.352	<.001
	***retro***	−4.387	−0.385	<.001
	***repeated***	−11.904	−1.044	<.001
**intervene-different**	***intervene-same***	−9.547	−0.837	<.001
	***retro***	3.150	0.276	0.003
	***repeated***	−6.801	−0.596	<.001
**intervene-same**	***retro***	11.970	1.050	<.001
	***repeated***	3.255	0.285	0.003
**retro**	***repeated***	−10.217	−0.896	<.001

*Note*. *P*-value adjusted for comparing a family of 15. Results are averaged over the levels of ‘Agent’.

#### 3.1.2. Linear mixed-effects model analyses

We implemented model selection using Complex Random Intercepts (CRI) models after observing that Maximal Model failed to converge and had a singular fit, even when optimization parameters were applied [39]. The CRI model included random intercepts for Subject, Subject*Gaze, Subject*Agent, Subject*Gaze*Agent and Trial ID. Trial ID captured variance attributable to trial-level features such as the specific direction of averted gaze. The random effect parameters defined in this model accounted for a substantial portion of variance as evidenced by the marginal *R^2^* (variance explained by fixed effects only; *R^2^* = .345) and conditional *R^2^* values (variance explained by both fixed and random effects; *R^2^* = .829). We found evidence for an effect of Gaze (*χ*2 = 132.91, *p* < .001), but no evidence for a significant effect of Agent (*χ*2 = 1.95, *p* = .163) or Gaze*Agent interaction (*χ*2 = 2.15, *p* = .829). Note that the *emmeans* package does not provide Holm-corrected confidence intervals. Hence, we computed the *z*-score associated with the rank of each Holm-corrected *p*-value and computed the upper and lower confidence intervals for each estimate separately (i.e. *β* ± *z*-score × SE).


GiveResponse∼Gaze+Agent+Gaze:Agent+(1|Sub)+(1|Sub:Gaze)+(1|Sub:Agent)+(1|Sub:Gaze:Agent)+(1|TrialID)

As summarized in table 6, Holm-corrected post hoc comparisons revealed significant differences between *all* Gaze condition pair-wise comparisons.

**Table 6.** Holm-corrected post hoc comparisons for Gaze condition effect.

		*β*	LCL_holm_	UCL_holm_	SE	*z*-ratio	*p*_holm_
**none**	***early***	−1.56	−2.40	−0.72	.336	−4.65	<.001
	***intervene-different***	−3.52	−4.45	−2.58	.337	−10.44	<.001
	***intervene-same***	−7.46	−8.56	−6.37	.373	−20.02	<.001
	***retro***	−2.83	−3.73	−1.93	.333	−8.49	<.001
	***repeated***	−6.38	−7.43	−5.33	.361	−17.70	<.001
**early**	***intervene-different***	−1.96	−2.83	−1.09	.337	−5.81	<.001
	***intervene-same***	−5.90	−6.98	−4.82	.372	−15.85	<.001
	***retro***	−1.27	−2.07	−0.47	.333	−3.81	<.001
	***repeated***	−4.82	−5.85	−3.79	.360	−13.38	<.001
**intervene-different**	***intervene-same***	−3.94	−4.99	−2.90	.373	−10.58	<.001
	***retro***	0.69	0.03	1.34	.334	2.06	.040
	***repeated***	−2.86	−3.81	−1.91	.361	−7.93	<.001
**intervene-same**	***retro***	4.63	3.58	5.68	.370	12.53	<.001
	***repeated***	1.08	0.20	1.96	.393	2.76	.012
**retro**	***repeated***	−3.55	−4.53	−2.57	.357	−9.93	<.001

*Note*. The *p*-values were adjusted for comparing a family of 15. Results are averaged over the levels of ‘Agent’.

### 3.2. Reaction times

In our pre-registration, we indicated an intention to explore whether differences in ‘Give’ frequencies across Gaze conditions were accompanied by differences in RTs when making ‘Give’ or ‘No Give’ decisions, assuming that shorter/longer RTs would index less/more certainty about the communicativeness of the agent’s gaze behaviour, respectively. We implemented the same LME analysis, as outlined above, adding a fixed effect for Response Type (i.e. ‘Give’ or ‘No Give’) to determine whether RTs varied as a function of condition and the type of response made.

Consistent with our LME analysis approach outlined above, we implemented a CRI model. The full CRI model returned a singular fit, and so we sequentially removed random intercepts with the least (i.e. near zero) variance until the model converged. This included the removal of two random intercepts, including (1|Sub:Gaze:Agent) and then (1|Sub:Gaze); resulting in the following CRI model. The random effect parameters defined in this model accounted for a substantial portion of variance as evidenced by the marginal *R^2^* (variance explained by fixed effects only; *R^2^* = .082) and conditional *R^2^* values (variance explained by both fixed and random effects; *R^2^* = .403).

We found evidence for an effect of Gaze (*F*_(5,53.92)_=18.87, *p* < .001), Response (*F*_(1,137.76)_=7.23, *p* = .008), and a Gaze*Response interaction (*F*_(5,1073.62)_=62.59, *p* < .001). Once again, we found no evidence for an effect of Agent or associated interactions (all *p*s>.156).


ReactionTime Gaze∗Agent∗Response+(1|Sub)+(1|Sub:Agent)+(1|Sub:Response)+(1|Sub:Gaze:Response)+(1|Sub:Agent:Response)+(1|Sub:Gaze:Agent:Response)+(1|TrialID)

As summarized in table 7, we conducted Holm-corrected post hoc comparisons to confirm which gaze conditions exhibited significant RT differences between ‘Give’ and ‘No Give’ responses. We found that participants were significantly faster to respond with a ‘No Give’ than ‘Give’ response in the two least communicative conditions (see frequency analysis above); that is the None (*β* = -.026, SE = .037, *t* = −7.09, *p* < .001) and Early (*β* = -.127, SE = .037, *t* = −3.45, *p* = .017) gaze conditions, suggesting that participants rapidly and confidently evaluated the agent in these trials as displaying non-communicative behaviour. Conversely, the opposite tendency was observed in the two most communicative conditions; that is the Repeated (*β* = .327, SE = .043, *t* = 7.56, *p* < .001) and Intervene-Same (*β* = .551, SE = .051, *t* = −10.75, *p* < .001) gaze conditions, were characterized by faster RTs for ‘Give’ than ‘No Give’. This suggests that participants in these conditions readily characterized the agent as displaying communicative behaviour. We found no evidence for significant differences between ‘Give’ and ‘No Give’ responses within the Retro and Intervene-Different conditions (all *p*s > .739). This likely reflects that these conditions were more ambiguous in their signalling of communicative intent. RT data by condition, and collapsed across agent, is summarized in figure 3B.

**Table 7.** Holm-corrected post hoc response comparisons by Gaze condition.

	‘Give’ *M*(SD)	‘No Give’ *M*(SD)	*β*	LCL_holm_	UCL_holm_	SE	*t*-ratio	*p*_holm_
***none***	897.12 (376.47)	696.69 (295.87)	−.261	−.384	−.138	.037	−7.09	<.001
***early***	1025.36 (416.38)	937.17 (418.07)	−.127	−.243	−.012	.037	−3.45	.017
***intervene-different***	869.84 (344.71)	921.72 (397.82)	0.010	−.095	.115	.037	0.27	.785
***intervene-same***	553.54 (137.95)	1069.77 (607.67)	.551	.378	.724	.051	10.75	<.001
***retro***	835.75 (361.66)	786.85 (368.84)	−.077	−.187	.034	.036	−2.11	.739
***repeated***	646.54 (145.88)	1017.29 (513.91)	.327	.183	.472	.043	7.56	<.001

*Note*. The *p*-values were adjusted for comparing a family of 15. Results were averaged over the levels of ‘Agent’.

### 3.3. Godspeed comparisons by agent (human versus robot)

Although we found no evidence for behavioural differences between the two agent conditions, we did find significant within-subjects differences in subjective evaluations made about these agents across three of the five Godspeed subscales [36]. Specifically, participants rated the Human agent significantly higher for Anthropomorphism and Animacy than the Robot agent, but lower for Likeability, with no evidence for significant differences for ratings on Perceived Intelligence and Safety (see table 8 for a complete summary of descriptive and test statistics).

**Table 8.** Godspeed ratings of human and robot agent.

	*M*	SD	W	*p*	rank-biserial correlation	95% CI for rank-biserial correlation	
**anthropomorphism**						**lower**	**upper**
***human***	2.682	1.076	5121.00	<.001	0.083	−.109	0.270
***robot***	2.251	0.914					
**animacy**							
***human***	2.869	0.996	5121.00	<.001	0.083	−.109	0.270
***robot***	2.611	0.812					
**likability**							
***human***	3.327	0.804	2437.00	0.008	−.484	−.618	−.324
***robot***	3.496	0.771					
**perceived intelligence**							
***human***	3.365	0.694	2210.50	0.092	−.532	−.656	−.380
***robot***	3.463	0.736					
**perceived safety**							
***human***	3.015	0.575	1293.50	0.052	−.726	−.805	−.622
***robot***	3.135	0.605					

*Note*. Test statistics summarize Wilcoxon signed-rank comparisons between the human and robot agent conditions for each of the Godspeed scales.

## 4. Discussion

Evaluating the communication intentions of others is important for effectively navigating reciprocal communication and coordination during social interactions. People are particularly sensitive to eye contact (i.e. direct gaze) displays, which signal an interlocutor’s readiness or intention to communicate and interact [8,10,11,14]. The current study conducted the first systematic investigation of the perceptual properties that influence the extent to which eye contact is interpreted as communicative. Specifically, we interrogated perceptions of communicative intent across six gaze conditions that manipulated the temporal sequence of eye contact within dynamic eye movement displays. We hypothesized that more recent and frequent eye contact displays in a dynamic sequence of eye movements would be perceived as more communicative than sequences with earlier, fewer or no eye contact displays at all. The results of our study partially supported these hypotheses. Specifically, we found that the temporal context of eye contact—defined as the combination of gaze shifts before and after eye contact—played a crucial role in shaping perceptions of communicative intent. The most communicative conditions were those where eye contact occurred between two averted gaze shifts directed at the same object (Intervene-Same) and where eye contact was repeated before and after an averted gaze shift (Repeated). Contrary to our hypotheses, the Intervene-Same condition was perceived as the most communicative, significantly more than the Retrospective Eye Contact condition. These findings suggest that it is not merely the temporal recency or frequency of eye contact that matters, but the specific sequence and combination of gaze behaviours that create a meaningful context for interpreting communicative intent. We discuss these findings in more detail in the following section.

Overall, we found robust differences between all gaze conditions, reflecting that it is the surrounding context of averted gaze before or after an eye contact display that is more critical than its temporal recency or frequency in signalling an agent’s communicative intent.

### 4.1. The temporal effects of eye contact

We present evidence that any eye contact display, irrespective of its temporal sequence in a dynamic series of eye movements, increased perceptions of communicative intent, compared with dynamic gaze displays without eye contact. Not only were participants less likely to perceive communicative intent when their partner displayed no eye contact, but they were also significantly faster to make this decision. On the other hand, people were most likely to perceive communicative intent when the agent conveyed eye contact between two averted gaze shifts at the same object (Intervene-Same), followed by instances where the agent conveyed two eye contact displays before and after a single averted gaze shift at an object (Repeated). In these conditions, participants were also significantly faster to execute ‘Give’ responses than ‘No Give’ responses; again, demonstrating increased certainty in the evaluation of the agent’s behaviour as communicative in these trials.

Our findings reveal that it is the temporal *context* of eye contact (i.e. the combination of eye movements made before and after eye contact), rather than the temporal position or recency of eye contact displays that matters most. This is most clearly shown by the fact that the Retro condition, in which the gaze sequence ends with eye contact, was one of the most ambiguous conditions for signalling communicative intent. Furthermore, the Intervene-Different and Intervene-Same conditions present eye contact in the very same temporal position but differ in the surrounding context of averted gaze displays. Our finding of significantly stronger perceptions of communicative intent in the Intervene-Same condition (i.e., eye contact flanked by gaze shifts at the same location), than the Intervene-Different condition (i.e. eye contact flanked by gaze shifts at different locations) shows that the repetitive display of averted gaze, in combination with eye contact, can signal intentionality or interest in a potential locus of joint attention. This aligns with previous joint attention experimental research, which found that people were significantly faster to respond to gaze-cued joint attention bids that followed eye contact and a preceding gaze shift towards the joint attention location than when random gaze shifts were followed by eye contact and subsequent joint attention bids [19–21]. It also aligns with findings that repetitive actions are perceived as efficient signals for communicative action [24].

What remains unclear is whether repeated averted gaze displays towards an object increase perceptions of communicative intent independent of eye contact; that is, when an agent repeatedly looks at an object before and after looking at another object, instead of making eye contact. Future work that separately manipulates these perceptual factors of averted gaze repetition, with and without eye contact, would help fully elucidate their independent and shared influence on perceptions of communicative intent. Future research should also assess the role of eye contact duration, another perceptual feature that may influence the potency of communication signals. We know anecdotally and empirically that the duration of eye contact influences arousal and comfort [44,45], but the role of eye contact duration on perceptions of communication (i.e. intention, readiness) has not yet been investigated.

Another interesting finding in the current study was the limited influence early eye contact displays had on evaluations of communicative intent. One possible explanation for the low rates of perceived communicative intent in the Early Eye Contact condition is that some participants may have considered some initial eye movements necessary for the agent to assess block availability. As such, they may have dismissed early eye contact displays as irrelevant. This possibility could be illuminated in future implementations of this paradigm by making it explicitly clear in the instructions that block availability is immediately apparent to the agent from the beginning of the trial. However, this explanation is unlikely given that ‘Give’ rates were high for Repeated Eye Contact, where the agent only averted gaze to one location (i.e. does not overtly fixate multiple blocks to ascertain availability). A more likely explanation for the observed effects is that Early Eye Contact was not potent in signalling communicative intent because eye contact and the final gaze shift was intervened by another gaze shift to a different location, hence reducing the relevance of the initial eye contact and final gaze shift.

It would also be of value for future work to examine how gaze dynamics influence the detection of communication opportunities and joint attention responsivity in more dynamic, multi-gestural contexts. It would be particularly valuable to understand how eye contact is interpreted when other explicit communication behaviours are present, such as waving, hand-pointing, physical touch or ostensive facial expressions (e.g. raised eyebrows). Indeed, Caruana and colleagues have shown in several studies that human dyads naturally attend to and use the gaze information displayed by their partner during tasks that explicitly require coordination using hand-pointing gestures [6,7]. However, there was marked variation across dyads with respect to the reliability of gaze displays, and thus the extent to which they were attended to. Future studies that manipulate the potency of eye contact displays in multi-gestural interactions would inform whether the integration of communicative cues across multiple communicative modalities can be optimized using ostensive signals such as eye contact. By programmatically examining the individual and interacting role these cues have on the perception of communicative intent, this line of research will critically inform how to design the communicative behaviours of artificial agents so that they promote natural and intuitive interaction with human users.

Finally, while the current study comprised an ethnically diverse sample, approximately half the sample was white. As such, our sample likely represents largely Western cultural norms, and future work is needed to fully assess whether the perception of gaze, from both human and robotic agents, generalizes to people of all cultural backgrounds. It has been suggested that eye contact behaviour is reduced among people from Eastern cultural backgrounds, given cultural norms that promote the avoidance of eye contact as a sign of respect [46]. However, more recent empirical evidence has failed to support such claims. For instance, recent work examining dyadic interactions using head-mounted eye-tracking found the opposite to be true—with East Asians engaging in eye contact more frequently and for longer instances than Western Caucasians during a storytelling game [47]. Nevertheless, future research would benefit from a more targeted approach to examining cultural differences in gaze perception and interpretation in interactive contexts, both with human and artificial interlocutors.

### 4.2. Agent appearance effects

A secondary aim of the current study was to explore whether the perceptual effects of eye contact on communication perception generalized or differed when signalled by agents that appeared human-like or as robots. We found robust Bayesian evidence for commensurate effects of gaze across both agent conditions. This is particularly surprising given that the agents not only differed in their visual anthropomorphism—but also in terms of the low-level perceptual features of their eyes, as our stimuli were selected to accurately model real human or robot agents. Our findings suggest that the dynamics that influence the perception of gaze as communicative likely generalize across agents that vary in their human-likeness; at least when the agent’s eyes anatomically resemble those of humans. This is broadly consistent with the gaze-cueing literature, which has documented robust cueing effects across experiments implementing gaze stimuli with varying degrees of ecological validity, but that use stimuli—including robot faces—that have eyes that anatomically resemble those of humans [25,27]. Contrastingly, studies that have shown differences in the influence of gaze on intention evaluations between human and robotic agents have examined robot stimuli with markedly less human-like eyes, such as the NAO (fixed eye position) or Baxter robots (schematic eyes on a screen) [29,30]. As such, further work is needed to systematically extend this line of inquiry to examine how gaze dynamics influence communication perception—and coordination—with artificial agents that vary in their human-likeness, in both virtual or screen-based agents and physically embodied robots. Such work is critical for informing the human-centred design of intuitive social robots that promote collaboration and trust.

It is also possible that robotic agents that appear more anthropomorphic may lead observers to implicitly adopt an ‘intentional stance’ towards these agents [48]. That is, people may be more likely to evaluate and respond to the agents as if they are intentional and sentient entities (i.e. like humans). Therefore, to fully understand the extent to which the knowledge from the current study can be used to inform the design of gaze behaviour in robotic agents, we must next determine whether evaluations of gaze are also influenced by beliefs about the agent’s intentionality, sentience and intelligence. This is critical for several reasons. First, there is currently huge variation in the expectations people have about robots, in terms of their autonomous capabilities [49]; likely influenced by varied depictions of robots in popular culture and the rapid developments currently being made in artificial intelligence, to which people have varying degrees of understanding and exposure. Second, we know, from previous work using subjective, behavioural and neural measures of gaze processing, that the social significance of eye gaze is evaluated differently depending on whether the observer believes an agent is controlled by an intentional human, or a pre-programmed computer algorithm [50–53]. In the current study, conducted online, participants likely assumed both agents were artificial (i.e. neither intentional nor intelligent). It would be of value to empirically evaluate whether perceptions of communicative intent in this task would be elevated across all conditions if people explicitly believe they are observing the live eye movements of another human or sentient agent, and further, whether this intentional stance belief effect would interact with the human-like or robotic appearance of the agent.

### 4.3. Implications and conclusion

This study presents the first systematic and objective interrogation of the temporal features of dynamic eye contact displays that influence the potency of communication signals, as displayed by both human-like and robotic agents. The knowledge delivered by this line of inquiry will help define specific models of social information processing during face-to-face interactions that can inform how to engineer communicative behaviours in artificial agents (e.g. virtual avatars, social robots). This human-centred approach to artificial agent design is key to realizing intuitive and acceptable artificial agents for applied human–robot interactions, promoting their usefulness as tools for collaboration, companionship or training. Furthermore, identifying the eye contact signals that we *implicitly* use during social interactions may help identify *explicit* principles for effective non-verbal communication between human interlocutors. Such principles are key to guiding social-cognitive training around how to effectively communicate with others in social contexts that rely on non-verbal communication (e.g. competitive sports, military operations, loud environments), for those who rely on non-verbal communication (e.g. hearing-impaired), and for those who might find non-verbal and gaze-based communication difficult or uncomfortable in certain contexts (e.g. autistic people).

## Data Availability

Pre-registration: The hypotheses, methods and analyses were pre-registered [54] on 23/06/2023, prior to data collection. Analyses were conducted as pre-registered. However, we additionally conducted analyses using linear mixed effects modelling (not pre-registered) for reasons detailed in the exploratory analyses of RT data did deviate from pre-registration for reasons clearly explained under ‘§2.4.2’. Materials: All study materials are publicly available [54]. Data: All primary data are publicly available [54]. Analysis scripts: All analysis scripts are publicly available [54].

## Footnotes

^1^
Exploratory correlation analyses did reveal a marginally significant positive association between CATI-Communication subscale scores and ‘Give’ frequencies in the Retro gaze condition only (described below; *r* = .17; *p* = .046; uncorrected for multiple comparisons). This was characterized by a stronger tendency to perceive communicative intent among people with greater self-reported communication difficulties. The reliability of this effect requires prospective investigation.
